# Prediction of Cerebral Amyloid Pathology Based on Plasma Amyloid and Tau Related Markers

**DOI:** 10.3389/fneur.2021.619388

**Published:** 2021-10-04

**Authors:** Ting-Bin Chen, Kun-Ju Lin, Szu-Ying Lin, Yi-Jung Lee, Yi-Cheng Lin, Chen-Yu Wang, Jun-Peng Chen, Pei-Ning Wang

**Affiliations:** ^1^Department of Neurology, Neurological Institute, Taichung Veterans General Hospital, Taichung, Taiwan; ^2^Dementia Center, Taichung Veterans General Hospital, Taichung, Taiwan; ^3^Center for Geriatrics and Gerontology, Taichung Veterans General Hospital, Taichung, Taiwan; ^4^Department of Nuclear Medicine and Molecular Imaging Center, Linkou Chang Gung Memorial Hospital, Taoyuan, Taiwan; ^5^Healthy Aging Research Center and Department of Medical Imaging and Radiological Sciences, College of Medicine, Chang Gung University, Taoyuan, Taiwan; ^6^Taipei Municipal Gan-Dau Hospital, Taipei, Taiwan; ^7^Division of Neurology, Department of Medicine, Taipei City Hospital Renai Branch, Taipei, Taiwan; ^8^School of Life Sciences, Institute of Neuroscience, National Yang-Ming University, Taipei, Taiwan; ^9^Division of General Neurology, Department of Neurological Institute, Taipei Veterans General Hospital, Taipei, Taiwan; ^10^Biostatistics Task Force of Taichung Veterans General Hospital, Taichung, Taiwan; ^11^Faculty of Medicine, School of Medicine, National Yang Ming Chiao Tung University, Taipei, Taiwan; ^12^Brain Research Center, National Yang Ming Chiao Tung University, Taipei, Taiwan

**Keywords:** pyroglutamate, tau, β-amyloid, Alzheimer's disease, predictor

## Abstract

**Background and Purpose:** Pyroglutamate-modified β-amyloid peptide (Aβ_pE_) is crucial for AD pathophysiological process. The potential associations of plasma Aβ_pE_ and total tau (t-tau) with brain Aβ burden and cognitive performance remain to be clarified.

**Methods:** Forty-six subjects with unimpaired cognition, mild cognitive impairment, or very mild dementia were enrolled. Plasma levels of Aβ_pE3−40_, t-tau, and Aβ42 were quantified by immunomagnetic reduction (IMR) assays. We analyzed individual and combined biomarker correlations with neuropsychological scores and Aβ positivity determined by ^18^F-florbetapir positron emission tomography (PET).

**Results:** Both plasma Aβ_pE3−40_ levels and Aβ_pE3−40_/t-tau ratios correlated negatively with short-term memory and global cognition scores, while correlating positively with PET standardized uptake value ratios (SUVRs). Among the biomarkers analyzed, the combination of Aβ_pE3−40_ in a ratio with t-tau had the best discriminatory ability for Aβ PET positivity. Likewise, logistic regression analysis showed that Aβ_pE3−40_/t-tau was a highly robust predictor of Aβ PET positivity after controlling for relevant demographic covariates.

**Conclusion:** Plasma Aβ_pE3−40_/t-tau ratios correlate with cognitive function and cerebral Aβ burden. The suitability of Aβ_pE3−40_/t-tau as a candidate clinical biomarker of AD pathology in the brain should be examined further in larger studies.

## Introduction

Alzheimer's disease (AD) underlies a major unmet medical need in routine clinical practice and casts a considerable burden on patients, caregivers, and societies. The neuropathological characteristics of AD include neuronal loss, β-amyloid (Aβ) plaques, neurofibrillary tangles, and synaptic loss (1, 2).

The main Aβ variants detected in the human brain are Aβ40 and Aβ42. They are found together with N-terminal truncated forms of these variants (Aβn-40/42), which have been shown to have pyroglutamate modifications, yielding a distinct pyroglutamate-modified Aβ peptide (Aβ_pE_) (3–6). The Aβ_pE_ peptide has been identified in early-stage AD, even before clinical symptoms are apparent, implicating it as a potential seeding molecule that may enable pathological Aβ aggregate formation and triggering hyperphosphorylation of tau (6–11). Aβ_pE_ is formed when full-length Aβ peptides undergo truncation at the N-terminal glutamate and subsequent dehydration catalyzed by glutaminyl cyclase (12, 13). Compared to full-length peptides, Aβ_pE_ displays greater β-sheet content, hydrophobicity, aggregation propensity, deposition, amyloidogeneity, resistance to enzymatic degradation, and neuronal toxicity (9, 14–17). In addition, Aβ_pE_ has been reported to be closely associated with cognitive decline, disease progression, and cerebral amyloidosis in AD (8, 10, 18, 19), suggesting that it may be a key driving force in AD pathogenesis.

Amyloidosis, a primary characteristic of AD pathology, can be detected by positron emission tomography (PET) imaging and cerebrospinal fluid (CSF) measures (20, 21). Both measures possess high diagnostic and prognostic value and may reveal changes many years prior to clinical onset of AD (22–25). Because PET scans are costly and have limited availability, they cannot be incorporated broadly into routine clinical assessments of cognitive impairment before initiation of AD treatment therapies. By contrast, although CSF sampling is less costly and more readily available, it is quite invasive. Neither the PET nor the CSF test is appropriate for population-based screening aimed at identifying high-risk individuals before symptom onset. Moreover, approximately 70% of study participants with clinical syndromes of AD dementia in AD prevention trials and around 25% of participants in AD drug trials did not have brain amyloidosis that was detectable by PET (26, 27). Thus, a minimally invasive measure, such as a blood test, that can reflect cerebral AD pathology accurately and reliably would have critical advantages for supporting clinical decisions regarding AD treatment planning and monitoring of patients during therapeutic trials.

Immunomagnetic reduction (IMR) assay is a relatively new ultrasensitive detection technology capable of quantifying biomarkers down to pg/mL levels. Previously, we observed a positive correlation of IMR-quantified plasma levels of a pyroglutamate cyclization variant of N-terminally truncated Aβ3–40 (Aβ_pE3−40_) with cerebral amyloidosis detected by PET imaging (18). In the present study, IMR was implemented to measure plasma levels of Aβ_pE3−40_, t-tau, and Aβ42. The primary aim of this work was to investigate associations of these plasma biomarkers, individually and in combination, with cognitive performance and to assess their utility for predicting Aβ PET positivity (+).

## Materials and Methods

### Subjects

Eligible subjects at Taipei Veterans General Hospital, Linkou Chang Gung Memorial Hospital, and Kaohsiung Chang Gung Memorial Hospital were enrolled through the Alzheimer's Disease Neuroimaging Initiative in Taiwan (T-ADNI). The local institutional review boards and the ethics committee of the three hospitals approved the data collection protocol. T-ADNI study was approved by the ethics committees of the three hospitals. The inclusion criteria were an age of 55 to 90 years and educational attainment of at least 6 years. Prior to testing, written informed consent was obtained from all participants and/or their legal guardians. All methods were performed in accordance with the relevant guidelines and regulations.

All participants were interviewed by experienced neurologists to collect information about their demographics, family history, and physical/neurological condition. Additionally, for each participant, neurologists obtained Hachinski ischemic score and vital signs. Blood was drawn for a hematology/chemistry panel and for vitamin B12s, thyroid-stimulating hormone, free thyroxine, and rapid plasma reagin syphilis tests.

A standard battery of neuropsychological tests was administered, including the Geriatric Depression Scale, Mini-Mental State Examination (MMSE) (28), Chinese Version Verbal Learning Test (29), Logical Memory subscale (story A) of the Chinese version of the Wechsler Memory Scale-III (30), 30-item Boston Naming Test (31), categorical Verbal Fluency Test (29), Trail-Making Test A and B (line) (32), and Clinical Dementia Rating Scale (CDR) (33). Only subjects with non-impaired cognition, defined by CDR score of 0, and patients in the prodromal stage or very mild dementia stage, defined by CDR score of 0.5, were invited to participate in this study.

Signs of cognitive impairment were assessed and non-AD etiologies (e.g., tumors, strokes, severe white matter disease, or inflammation) were excluded through a series of clinical interviews, a physical examination, screening laboratory tests, neuropsychological assessments, and brain magnetic resonance (MR) imaging. Exclusionary criteria included any history of major brain trauma, brain tumor, stroke, epilepsy, alcoholism, major psychiatric illness, or any other systemic diseases that might affect cognitive function. Diagnosis of amnestic mild cognitive impairment and probable AD were made on the basis of the core clinical criteria developed by the National Institute on Aging and the Alzheimer's Association's 2011 workgroup (NIA-AA) (34). The demographic characteristics of the study cohort, dichotomized according to Aβ PET results, are summarized in Table 1.

**Table 1:** Demographic and clinical characteristics of enrolled subjects (*N* = 46).

**Characteristic**	**Aβ PET – (*N* = 28)**	**Aβ PET+ (*N* = 18)**	***P***
Female/male ratio	11/17	11/7	>0.999
Age, years	69.5 (60.3–77.3)	72.0 (64.0–78.3)	0.373
Education, years	12.0 (9.0–15.0)	13.0 (11.3–16.0)	0.273
Clinical stage			0.005
HC	5	0	
aMCI	18	7	
AD	5	11	
ApoE e4 allele carrier	7.7%	25%	0.038
MMSE	27.5 (25.3–29.0)	23.5 (22.0–26.3)	0.001
CDR			0.072
0	5	0	
0.5	23	18	
Global SUVR	1.1 (1.0–1.2)	1.5 (1.4–1.7)	<0.001
Aβ_pE3−40_, fg/mL	42.3 (19.0–60.7)	65.7 (54.3–118.6)	0.003
t-tau, pg/mL	21.3 (13.9–30.6)	14.9 (13.2–18.3)	0.072
Aβ42, pg/mL	17.3 (14.8–19.4)	15.3 (14.9–16.5)	0.115
Aβ_pE3−40_/t-tau	1.72 (0.734–3.174)	4.39 (2.683–9.091)	0.001
Aβ42/t-tau	0.8 (0.7–1.0)	1.0 (0.7–1.1)	0.9

*PET, positron emission tomography; HC, healthy control; aMCI, amnestic mild cognitive impairment; AD, Alzheimer's disease; CDR, clinical dementia ranking; MMSE, mini–mental state examination; SUVR, standardized uptake value ratio*.

*Continuous variables, presented as median (interquartile range), were analyzed with Mann–Whitney U tests. Categorical variables, presented as number of subjects or percentage, were examined by a Chi-square test*.

### Collection and Preparation of Human Plasma Samples

An 8-ml non-fasting venous blood sample (K3 EDTA, lavender-top tube) was collected from each subject and then centrifuged (1,500–2,500 ×*g* for 15 min) within 1 h of the draw. The plasma was then aliquoted into cryotubes and stored at −20°C.

### IMR Measurements

Detailed descriptions of the IMR platform and validation of its accuracy have been published previously (18, 35). IMR reagents were selected based on epitope antigen/antibody affinity, ability to conjugate with MagQu magnetic Fe_3_O_4_ nanobeads, and ability to produce linear standard curves of quantitated magnetic signal reduction. Each type of reagent consists of magnetic nanoparticles dispersed in phosphate buffered saline (pH 7.2). By immobilizing functionalized monoclonal antibodies against Aβ37–42 (ABCAM, Cambridge, UK; ab34376) and t-tau protein (Sigma Aldrich; T9450) on the magnetic nanoparticles, two types of reagents were obtained. The antibody against Aβ_pE3−40_ was developed by Biogen Inc. The mean diameter of the antibody-functionalized magnetic nanoparticles was 50–60 nm. The magnetic concentration of each type of reagent was 12 mg-Fe/mL. Duplicated/paired measurements of Aβ42, Aβ_pE3−40_, and t-tau were performed for each plasma sample. We mixed 60-μl plasma samples with 60 μl of reagent (MF-AB2-0060 or MF-DEX-0060, respectively; MagQu) at room temperature in the Aβ42 and Aβ_pE3−40_ assays; for the t-tau assay, 40 μl of plasma with 80 μl of reagent (MF-TAU-0060; MagQu) were used. Magnetic signal changes during the course of interactions between antigens and antibody-functionalized magnetic nanoparticles were detected by an IMR reader (XacPro-S, MagQu) and expressed as percentage reductions in immunomagnetic signal, which were then converted to a sample concentration based on reference to standard curves of the respective analytes. The ratio of the reduction to the alternative-current (ac) magnetic signal of reagent before incubation is expressed as follows:


$$\begin{array}{l} IMR\left(\%\right)=\frac{\chi_{ac,o}-\chi_{ac,\phi }}{\chi_{ac,o}}\times 100\%, \end{array}$$

where χ_ac,o_ and χ_ac,ϕ_ are the ac magnetic signals of reagent before and after incubation. For each reported IMR (%) in this study, an averaged value of duplicated IMR measurements was calculated. The standard deviations of all duplicated plasma analyte measurements were <15%. The reported analyte concentrations for each sample are means of the paired measurements.

### Analysis of ApoE Genotypes

*ApoE* genotyping was determined by polymerase chain reaction amplification and DNA sequencing (36). Participants with one or two ε4 alleles were defined as ε4 carriers.

### Aβ PET Imaging Data Acquisition

All Aβ PET imaging scans were performed on a Biograph mCT PET/CT scanner (GE Healthcare, Milwaukee, WI) at a single site (Linkou Chang Gung Memorial Hospital) as described in detail elsewhere (37, 38). Briefly, the scan commenced with a 10-min acquisition period (two 5-min frames) beginning 50-min after a 10-mCi injection of ^18^F-florbetapir tracer. Each image was obtained with the application of a three-dimensional ordered subset expectation maximization reconstruction algorithm (four iterations, 24 subsets; Gaussian filter: 2 mm; zoom: three) with computed tomography-based attenuation correction, as well as scatter and random corrections, with a matrix size of 400 × 400 × 148 and a voxel size of 0.68 × 0.68 × 1.5 mm^3^. To achieve useful anatomical information and facilitate co-registration with PET images, structural MR scans were obtained for all subjects with a uniform scanning protocol that minimizes and accounts for between-site differences in MR imaging systems.

### Aβ PET Imaging Processing

PET imaging data were processed and analyzed in PMOD software (version 3.7, PMOD Technologies Ltd., Zurich, Switzerland), including MR-based spatial normalization to the Montreal Neurological Institute MRI template. We selected seven volumes of interest: frontal, anterior cingulate, posterior cingulate, precuneus, parietal, occipital, and temporal cortical areas. We calculated regional standardized uptake value ratios (SUVRs) for each volume of interest, using the whole cerebellum as a reference region, and then averaged the SUVRs for the seven volumes of interest to yield an estimated global cortical SUVR value for further analysis.

All the PET images were interpreted by an experienced nuclear medicine physician (Kun-Ju Lin) who did not have access to clinical data. Aβ burden was graded on a five-point visual scale, from 0, indicating no tracer retention in cortical gray matter, to 4, denoting high levels of cortical amyloid accumulation. Scores of 0 or 1 were categorized as Aβ PET negativity (–) and scores of 2–4 were categorized as Aβ PET+ (39).

### Statistical Methods

All statistical analyses were performed in SPSS version 22.0 for Windows (SPSS Inc., Chicago, IL, USA). A *P* < 0.05 was considered significant. All variables were analyzed by non-parametric methods. For continuous variables, differences between Aβ PET- group and Aβ PET+ group were detected with Mann-Whitney U tests. For categorical variables, Chi-square tests were used. We generated neurocognitive numeric composite z-scores by calculating individual z-scores for each test and then averaging them across the cognitive test set. The constituents of the composite z-scores were as follows: short-term memory [Chinese Version Verbal Learning Test and Logical Memory subscale (story A) of the Chinese Version Wechsler Memory Scale-III], semantic memory [Boston Naming Test (total)]; executive function [Trail-Making Test-A/B (line) and categorical Verbal Fluency Test (animal)]; global cognition [short-term memory test, semantic memory test, executive function test, and the MMSE]. Spearman's rank coefficients were calculated to determine correlation of plasma biomarker levels with estimated global cortical SUVR and domains of cognitive performance. Receiver operating characteristic (ROC) and area under the curve (AUC) analyses were performed to define cut-off points for each biomarker analyte or their ratios to further characterize discriminatory properties between the Aβ PET- and Aβ PET+ groups. Finally, logistic regression modeling was performed to investigate the predictive power of biomarker levels for Aβ PET+, in terms of odds ratios (ORs) and 95% confidence interval (CIs), with and without adjusting for age, sex, and ApoE ε4 carrier status.

## Results

### Demographic Data

A total of 46 subjects were enrolled in the study and divided into Aβ PET- and Aβ PET+ groups. Their demographic characteristics and neurocognitive scores are presented in Table 1. There were no significant between-group differences in age, sex, years of education, clinical stage or CDR scores, nor in plasma biomarker levels of t-tau, Aβ42, or Aβ42/t-tau. Compared to the Aβ PET- group, the Aβ PET+ group had more ApoE ε4 carriers (25% vs. 7.7%, *p* = 0.038), poorer performance on MMSE (23.5 vs. 27.5, *p* = 0.001), higher Aβ PET SUVR (1.5 vs. 1.1, *p* < 0.001), and higher levels of plasma Aβ_pE3−40_ (65.7 vs. 42.3, *p* = 0.003) and Aβ_pE3−40_/t-tau ratio (4.39 vs. 1.72, *p* = 0.001).

### Association of Plasma Biomarkers With Aβ Burden and Cognitive Performance

**Figure 1:**
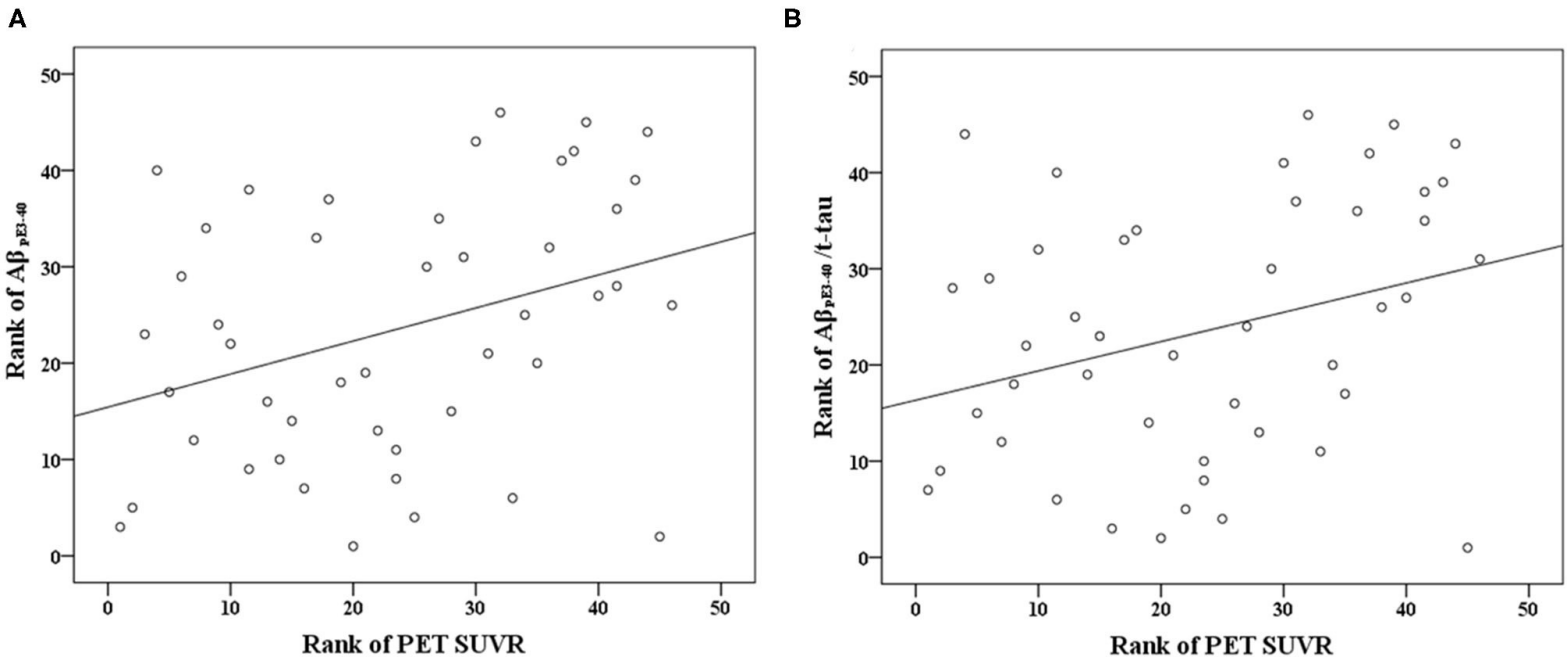
Scatterplots of the associations between Aβ PET SUVR and plasma biomarker levels (pg/mL) of **(A)** Aβ_pE3−40_ and **(B)** Aβ_pE3−40_/t-tau.

Aβ_pE3−40_ level (r = 0.343, *p* < 0.005), and Aβ_pE3−40_/t-tau ratio values (r = 0.305, *p* < 0.005) correlated directly with Aβ PET SUVRs (Table 2; Figure 1). Short-term memory scores correlated negatively with Aβ_pE3−40_ (r = −0.481, *p* < 0.001) and Aβ_pE3−40_/t-tau values (r = −0.483, *p* < 0.001), but positively with t-tau (r = 0.3, *p* < 0.005) and Aβ42 levels (r = 0.391, *p* < 0.001). Semantic memory scores were found to correlate positively with Aβ42 levels (r = 0.353, *p* < 0.005), while executive function scores correlated positively with Aβ_pE3−40_/t-tau values (r = 0.359, *p* < 0.005). Global cognition scores correlated negatively with Aβ_pE3−40_ (r = −0.337, *p* < 0.005) and Aβ_pE3−40_/t-tau values (r = −0.343, *p* < 0.005) while correlating positively with Aβ42 levels (r = 0.379, *p* < 0.005).

**Table 2:** Correlation of plasma biomarkers with Aβ burden and cognitive performance (*N* = 46).

**Aβ PET biomarker**	**Spearman r**_*****s*****_				
	**SUVR**	**Short-term memory**	**Semantic memory**	**Executive function**	**Global cognition**
Aβ_pE3−40_	0.343^*^	−0.481^**^	−0.084	0.268	−0.337^*^
t-tau	−0.041	0.300^*^	0.286	−0.248	0.305
Aβ42	−0.007	0.391^**^	0.353^*^	−0.216	0.379^*^
Aβ_pE3−40_/t-tau	0.305^*^	−0.483^**^	−0.140	0.359^*^	−0.343^*^
Aβ42/t-tau	0.073	−0.261	−0.236	0.229	−0.287

*SUVR, standardized uptake value ratio*.

* *p < 0.05*,

** *p < 0.01*.

### ROC Analysis for Prediction of Aβ PET Positivity

ROC-AUC analyses aimed at determining plasma biomarker discriminatory cut-off values yielded the following optimal significant cut-off values for the differentiation of Aβ PET– and Aβ PET+ groups: 55.45 fg/mL for Aβ_pE3−40_; 2.85 for Aβ_pE3−40_/t-tau; and 0.8 for Aβ42/t-tau. The combination of Aβ_pE3−40_ with t-tau in a Aβ_pE3−40_/t-tau ratio had a greater AUC than individual biomarkers (i.e., Aβ_pE3−40_, t-tau, and Aβ42) and the composite Aβ42/t-tau ratio. Detailed AUCs, cut-off values, sensitivities, and specificities are reported in Table 3.

**Table 3:** ROC analysis for identifying Aβ PET positivity (*N* = 46).

**Variable**	**AUC**	**(95% CI)**	***P***	**Optimal cutoff**	**Sensitivity (%)**	**Specificity (%)**
Aβ_pE3−40_	0.81	(0.61–0.87)	0.001	>55.45	83.33	71.43
t-tau	0.66	(0.50–0.79)	0.056	≤ 19.10	83.33	57.14
Aβ42	0.64	(0.48–0.78)	0.098	≤ 16.27	77.78	60.71
Aβ_pE3−40_/t-tau	0.83	(0.64–0.89)	<0.001	>2.85	83.33	75.00
Aβ42/t-tau	0.67	(0.51–0.80)	0.046	>0.8	77.78	60.71

*AUC, area under the curve; ROC, receiver operator characteristic curve*.

### Association of Plasma Biomarkers With Aβ PET Positivity

The relationship between plasma biomarkers and Aβ PET positivity was examined through a logistic regression analysis with the aforementioned optimal cut-offs. All of the analyzed markers were found to be significantly associated with Aβ PET positivity risk in both the unadjusted and adjusted logistic regression modeling results (Table 4). All adjusted ORs (aORs) became more pronounced after controlling for age, sex, and ApoE ε4 carrier status. Participants with Aβ_pE3−40_ > 55.45 fg/mL, t-tau ≤ 19.10 pg/mL, Aβ42 ≤ 16.27 pg/mL, Aβ_pE3−40_/t-tau > 2.85, or Aβ42/t-tau > 0.8 were at increased risk for Aβ PET positivity (aOR, 95% CI, and *p*-values are reported in Table 4).

**Table 4:** Association of plasma biomarkers with Aβ PET positivity (*N* = 46).

**Plasma markers** **Cut-off groups**	**Unadjusted**			**Adjusted^a^**		
	**OR**	**95%CI**	***P***	**OR**	**95%CI**	***P***
Aβ_pE3−40_, fg/mL						
≤ 55.45	Reference			Reference		
>55.45	12.36	(2.36–64.64)	0.003	21.75	(2.38–198.79)	0.006
t-tau, pg/mL						
>19.10	Reference			Reference		
≤ 19.10	6.66	(1.56–25.0)	0.010	16.66	(1.96–100.2)	0.011
Aβ42, pg/mL						
>16.27	Reference			Reference		
≤ 16.27	5.55	(1.41–20.0)	0.014	16.66	(1.85–100.1)	0.012
Aβ_pE3−40_/t-tau						
≤ 2.85	Reference			Reference		
>2.85	10.50	(2.58–42.68)	0.001	33.98	(3.37–342.83)	0.003
Aβ42/t-tau						
≤ 0.8	Reference			Reference		
>0.8	5.41	(1.41–20.77)	0.014	18.97	(2.04–176.34)	0.010

*CI, confidence interval; OR, odds ratio*.

a *Adjusted for age, sex, and ApoE e4 allele status*.

## Discussion

The current study assessed the association of levels of AD-related biomarkers, especially Aβ_pE3−40_, quantified by IMR technology with cognitive performance as well as their predictive power in Aβ PET positivity. We found that participants with Aβ PET+ had higher levels of plasma Aβ_pE3−40_ and higher Aβ_pE3−40_/t-tau ratio values than participants with Aβ PET-. These elevated values correlated positively with PET analysis findings and correlated negatively with short-term memory and global cognition scores. Aβ_pE3−40_ alone had a high discriminatory ability, and consideration of Aβ_pE3−40_ together with t-tau (i.e., in Aβ_pE3−40_/t-tau) provided greater differential value than any of the individual biomarker values examined and the Aβ42/t-tau ratio. After adjusting for demographic covariates (age, sex, and ApoE ε4 carrier status), Aβ_pE3−40_/t-tau proved to be the strongest predictive biomarker for Aβ PET positivity. Collectively, these findings suggest that plasma Aβ_pE3−40_/t-tau may indeed be reflective of cerebral amyloid pathology. To the best of our knowledge, the current study is the first to report relationships among plasma t-tau level, plasma Aβ_pE3−40_ level, and brain Aβ accumulation revealed by *in vivo* PET.

In a previous study, we observed a positive association of Aβ_pE3−40_ levels with Aβ PET SUVRs (18). In this study, we affirmed those prior findings and further showed that Aβ_pE3−40_-related biomarkers were more closely related to brain Aβ accumulation revealed by Aβ PET than were Aβ42, t-tau, and Aβ42/t-tau values. These results are discordant with previous data obtained with ultrasensitive analytical assays—such as, xMAP, stable isotope labeling kinetics-mass spectrometry, single-molecule array, and immunoprecipitation mass spectrometry approaches—showing that lower plasma Aβ42 levels and Aβ42/Aβ40 ratios were associated with a higher brain Aβ burden revealed by Aβ PET over the normal–mild cognitive impairment–AD cognitive spectrum (40–48). This divergence of findings could be consequent to the critically discrepant roles of Aβ_pE_ and Aβ42 in AD pathophysiological processes. Aβ_pE_ has been considered to be a principal initiator of early-stage AD pathogenesis and has been shown to correlate with tau pathology and cognitive decline in AD (10, 12, 15, 19, 49), whereas plasma Aβ42 was found to be inversely correlated with cortical Aβ burden, likely due to impaired Aβ plaque clearance from the brain or sequestration and deposition of Aβ species within the brain. A recent animal study showed the evidence that impaired meningeal lymphatic drainage can exacerbate the neuroinflammatory response and enhancing meningeal lymphatic function could achieve better ability of monoclonal antibodies to clear Aβ aggregates (50). Together with a phase 2 trial revealing that donanemab, a monoclonal antibody against Aβ_pE_ epitope, could curb cognitive decline in early AD (51), we speculate that Aβ_pE3−40_ is a better plasma biomarker than Aβ42 for detection and monitoring of cerebral amyloidosis.

We found that Aβ_pE3−40_, Aβ42, and Aβ_pE3−40_/t-tau correlated significantly with short-term memory and global cognition performance measures, whereas t-tau and Aβ42/t-tau did not correlate well with any cognitive performance measures in our study. Aβ_pE3−40_ has specifically been found to be more cytotoxic to hippocampal and cortical neurons than Aβ40 and Aβ42 in animal studies (52, 53), highlighting the close relationship to hippocampal-dependent cognitive impairment. In addition, Aβ_pE3−40_ and Aβ_pE3−40_/t-tau showed modestly positive correlation with Aβ PET SUVRs. In a previous study, Aβ42/t-tau ratios were also reported to have no correlation with Aβ PET SUVRs, but have positive correlation with brain tau accumulation and longitudinal changes in hippocampal volume and cerebral glucose metabolism (54). Unlike Aβ42/Aβ40 and Aβ42/t-tau in other studies, Aβ_pE3−40_/t-tau modestly paralleled with the presence of cerebral amyloidosis in this study. Our findings of a positive relationship between Aβ42 level and cognitive function measures are consistent with the findings of prior studies in which ultrasensitive detection methods were used, affirming the supposition that cognitive decline may be associated with low Aβ42 levels (55, 56), but not t-tau levels. There have been discordant findings regarding Aβ42 and t-tau levels correlating closely or not at all with cognitive test scores (35, 55, 57–66), perhaps due to methodological differences related to study design and the quantitative methods used. Aβ_pE_ has been reported to trigger AD-related neuronal loss, neurodegeneration, neurological deficits, and cognitive decline and has been shown to have differential expression patterns between AD progression and normal aging (19, 67–69). Similar to the finding indicating that a modified version of tau (i.e., ultrasensitive blood immunoassay-detected tau phosphorylated at threonine 181) (70) appears to be a better marker of disease progression than t-tau, Aβ_pE_-related marker is a better biomarker of brain Aβ burden and cognitive decline than Aβ42 or t-tau, and may thus provide useful insights into brain functioning in the AD continuum.

Our ROC-AUC analyses indicated that Aβ_pE3−40_ (*p* = 0.001), Aβ_pE3−40_/t-tau (*p* < 0.001), and Aβ42/t-tau (*p* = 0.046) can be used to differentiate between individuals with Aβ PET+ and individuals with Aβ PET-. Among the singular biomarkers, only Aβ_pE3−40_ had a significant and moderate-to-high predictive ability. Between the two aforementioned composite biomarkers, Aβ_pE3−40_/t-tau yielded a higher AUC (0.83) as well as greater sensitivity (83.33%) and specificity (75.00%). Among the five biomarkers analyzed, Aβ_pE3−40_/t-tau exhibited the best discriminatory power. Combining markers reflective of two distinct underlying pathophysiological derangements did indeed lead to an incremental benefit to between-group differentiation. There are differential time courses of Aβ and tau changes in AD progression (24, 62). The better correspondence to Aβ PET findings for Aβ_pE_, relative to other singular markers, may reflect Aβ_pE_ being an earlier marker than Aβ42 or tau in AD pathogenesis (9). Although subjects with an early disease stage (i.e., healthy controls with Aβ PET+) were not enrolled in this study, it may be reasonable to pursue Aβ_pE3−40_/t-tau as a potential predictor of cerebral Aβ deposition in population-based screening for high-risk individuals before AD symptom onset.

After controlling for demographic covariates, all of the presently examined putative biomarkers had some ability to predict Aβ PET positivity, with the combination of Aβ_pE3−40_ with t-tau appearing to be particularly useful for reflecting disease state along the AD continuum. Our ROC-AUC analyses indicated that Aβ_pE3−40_/t-tau possessed higher AUC, sensitivity, and specificity than Aβ42/t-tau, supporting the notion that utilization of plasma Aβ_pE3−40_ and t-tau together is superior to considering absolute levels of individual peptides or Aβ42/t-tau ratio as markers of cerebral amyloidosis. In the face of the high cost and low accessibility of Aβ PET restricting wide use in clinical practice, Aβ_pE3−40_/t-tau may help support clinical decisions and be used as a clinical screening tool to rule out individuals who do not need costly Aβ PET scanning in scenarios other than confirmatory diagnosis.

All patients in this study were classified into amnestic-type MCI or AD based on the clinical diagnostic criteria proposed by the 2011 NIA-AA workgroup (34). We intended to identify potential plasma biomarkers with proper cutoff values indicating Aβ PET positivity over the normal–mild cognitive impairment–AD cognitive spectrum, rather than to correlate clinical diagnoses with Aβ PET positivity or to differentiate clinical groups by Aβ PET positivity.

The current study had some limitations. First, we could only infer a possible association of plasma Aβ_pE3−40_ and t-tau with imminent risk of Aβ PET positivity. Owing to small sample size and lack of Aβ+ healthy controls, further replication, particularly with larger samples and longer follow-up, is warranted to validate the predictive values obtained in this study and to clarify the temporal relationship of these variables with Aβ PET+. Second, the striatum was not included in the SUVR measurements for amyloid PET. Third, we did not account for comorbidity covariates that may affect plasma biomarker levels in our analyses. Finally, the relatively small sample size of our cohort limits the generalizability of the present findings.

## Conclusion

In conclusion, a dual-factor biomarker consisting of the ratio between Aβ_pE3−40_ and t-tau measures, each determined by an ultrasensitive and easy-to-implement IMR assessment, showed superior performance characteristics and was in concordance with PET analysis and cognitive function assessment results. Given that Aβ and tau have long been considered hallmarks of AD pathogenesis, it may be reasonable to pursue an Aβ_pE3−40_ and t-tau composite factor as a predictor of cerebral Aβ deposition. Aβ_pE3−40_/t-tau might be an effective and feasible candidate for blood-based screening of cerebral AD-related neuropathology aimed at identifying at-risk individuals that should be recommended for CSF and/or PET studies prior to clinical treatment planning. These findings must be interpreted with caution given the relatively small sample size.

## Data Availability Statement

The raw data supporting the conclusions of this article will be made available by the authors, without undue reservation.

## Ethics Statement

The studies involving human participants were reviewed and approved by Taipei Veterans General Hospital, Linkou Chang Gung Memorial Hospital, and Kaohsiung Chang Gung Memorial Hospital. The patients/participants provided their written informed consent to participate in this study.

## Author Contributions

T-BC and P-NW contributed to the conception and design of the study, acquisition of data, interpretation of the data, critical revision of the manuscript, and wrote the first draft of the manuscript. K-JL conducted PET imaging analysis. S-YL, Y-JL, Y-CL, and C-YW were involved in the collection and/or analysis of data. J-PC performed statistical analysis. All authors read and approved the submitted version.

## Funding

This study was carried out with financial support from the Brain Research Center, National Yang-Ming University from The Featured Areas Research Center Program within the framework of the Higher Education Sprout Project by the Ministry of Education (MOE) in Taiwan, the Taiwan Alzheimer's Disease Neuroimaging Initiative (T-ADNI) group, the National Science Council and the Ministry of Science and Technology, Taiwan (MOST 105-2325-B-182A-005-, MOST 108-2321-B-010-013-MY2, and MOST 110-2321-B-010-007), Taipei Veterans General Hospital (V107C-090, V108C-060), and Chang Gung Memorial Hospital (CMRPG3D1802).

## Conflict of Interest

The authors declare that the research was conducted in the absence of any commercial or financial relationships that could be construed as a potential conflict of interest.

## Publisher's Note

All claims expressed in this article are solely those of the authors and do not necessarily represent those of their affiliated organizations, or those of the publisher, the editors and the reviewers. Any product that may be evaluated in this article, or claim that may be made by its manufacturer, is not guaranteed or endorsed by the publisher.
